# Childhood Vaccine Acceptance and Refusal among Warao Amerindian Caregivers in Venezuela; A Qualitative Approach

**DOI:** 10.1371/journal.pone.0170227

**Published:** 2017-01-20

**Authors:** Jochem Burghouts, Berenice Del Nogal, Angimar Uriepero, Peter W. M. Hermans, Jacobus H. de Waard, Lilly M. Verhagen

**Affiliations:** 1 Laboratorio de Tuberculosis, Instituto de Biomedicina, Universidad Central de Venezuela, Caracas, Venezuela; 2 Department of Pediatrics, Hospital de Niños J.M. de los Ríos, Caracas, Venezuela; 3 Facultad de Medicina, Universidad Central de Venezuela, Caracas, Venezuela; 4 Laboratory of Pediatric Infectious Diseases, Department of Pediatrics, Radboud Institute for Molecular Life Sciences, Radboud University Medical Center, Nijmegen, The Netherlands; Public Health England, UNITED KINGDOM

## Abstract

**Objectives:**

Acceptance of childhood vaccination varies between societies, affecting worldwide vaccination coverage. Low coverage rates are common in indigenous populations where parents often choose not to vaccinate their children. We aimed to gain insight into reasons for vaccine acceptance or rejection among Warao Amerindians in Venezuela.

**Methods:**

Based on records of vaccine acceptance or refusal, in-depth interviews with 20 vaccine-accepting and 11 vaccine-declining caregivers were performed. Parents’ attitudes were explored using a qualitative approach.

**Results:**

Although Warao caregivers were generally in favor of vaccination, fear of side effects and the idea that young and sick children are too vulnerable to be vaccinated negatively affected vaccine acceptance. The importance assigned to side effects was related to the perception that these resembled symptoms/diseases of another origin and could thus harm the child. Religious beliefs or traditional healers did not influence the decision-making process.

**Conclusions:**

Parental vaccine acceptance requires educational programs on the preventive nature of vaccines in relation to local beliefs about health and disease. Attention needs to be directed at population-specific concerns, including explanation on the nature of and therapeutic options for side effects.

## Introduction

Immunization is a proven tool for prevention of some of the most deadly childhood diseases. However, vaccines are underutilized, especially in developing countries. Around 1.5 million children die each year from vaccine-preventable infectious diseases [1]. Suboptimal vaccine coverage rates are often observed in ethnic minorities [2–5]. The dynamics of vaccine uptake are complicated and depend on both social factors and cultural perceptions. This includes not only perceptions of vaccinations and diseases, but also perceptions of vulnerability and protection and the role of medicines in producing and maintaining health [6]. Qualitative and quantitative studies addressing concerns about vaccination often fail to provide recommendations for interventions [7].

Approximately 10 percent of the South American population consists of indigenous people and low vaccination coverage rates are observed in these populations [8, 9]. Although specific barriers to vaccination have been identified in indigenous South American populations [10], there is a paucity of qualitative studies exploring the knowledge, attitudes and practices surrounding these barriers.

The Expanded Program on Immunization (EPI) in Venezuela operates under the Extended Immunization Program (Programa Ampliado de Inmunizaciones) of the Ministry of Public Health (Ministerio de Salud Pública). The Venezuelan immunization schedule includes Bacille Calmette Guérin and hepatitis B vaccine at birth; the diphtheria, tetanus, pertussis, hepatitis B and polio (OPV) vaccine at 2, 4, 6 and 18 months and 5 years of age; measles, mumps and rubella vaccine at 12 months and 5 years and oral rotavirus vaccination at 2 and 4 months [1]. The most recent addition to the immunization schedule was the incorporation of the 13-valent pneumococcal conjugate vaccine (PCV13) in 2014. The Warao people residing in the Orinoco river Delta along the eastern coast of Venezuela are the second largest Venezuelan indigenous population. As long as vaccination teams have entered this area, they have experienced resistance against vaccination. Vaccine refusal results in low vaccination coverage rates despite the availability of mobile vaccination teams. Only around 25% of Warao children are fully immunized with the EPI vaccines and this is only 18% for the non-EPI vaccines. These coverage rates are two to three times lower compared with other parts of Venezuela [9]. This is of particular concern since the living conditions of the Warao population, as well as many other South American indigenous populations, expose them to multiple health risks. The lack of access to medical care, clean drinking water and food leads to high prevalence rates of infectious diseases. Because most Warao people do not have access to basic health services, there is a shortage of official morbidity and mortality rates. In 2011, a cross-sectional survey including interviews on child survival with 668 Warao women from 97 communities was performed. This work showed that of almost 4,000 reported life births an extraordinary high number of 1,245 children (32%) had died, most of whom (97%) were under five years of age. Infectious diseases were the main reported cause of death and together responsible for 85% of the reported deaths [11].

We took a qualitative approach to identify important themes in the decision-making process around childhood vaccination in indigenous caregivers in Venezuela. The objective of this study was to provide recommendations for the understanding and modification of parental beliefs to influence decisions regarding vaccination, thereby improving uptake.

## Methods

### Study area

**Fig 1:**
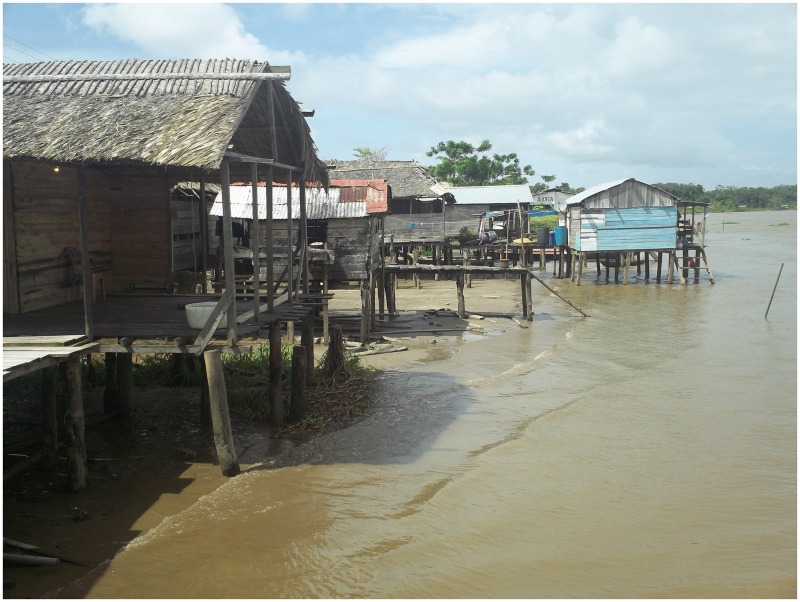
Picture of a typical Warao Amerindian village in the Orinoco Delta in Venezuela.

**Fig 2:**
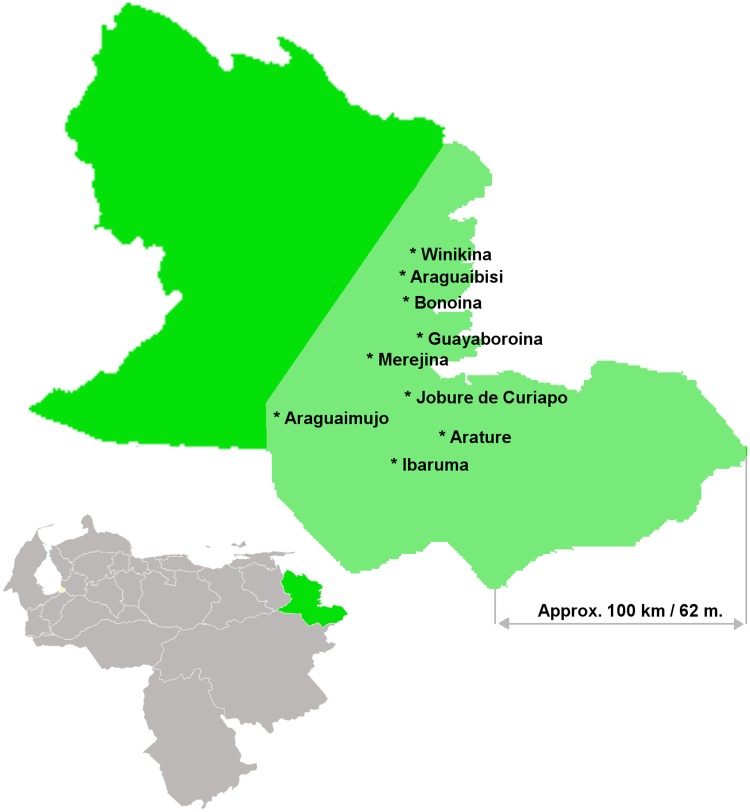
Simplified representation of Venezuela with the Orinoco Delta highlighted in green (bottom left) and the Antonio Diaz municipality within the Orinoco Delta (light green) with the nine study communities.

This study was conducted in the Orinoco Delta in Northeastern Venezuela. In this rural watery area, people live in small geographically isolated communities in wooden houses raised on piles along the Orinoco River banks (Fig 1). There is limited electricity supply, communication facilities or clean drinking water. The Orinoco Delta comprises circa 40,000 km^2^ and is inhabited by the Warao people, the second largest indigenous population in Venezuela. Almost one-third of Warao children die during childhood and respiratory tract infections are a major cause of death [11]. Our study was performed in the largest of the four municipalities comprising the Orinoco Delta, Antonio Diaz (Fig 2). In most Warao villages in Antonio Diaz a small poorly equipped health post is present. There is only one hospital with radiology and laboratory facilities in Antonio Diaz. Vaccination is generally carried out by regional mobile vaccination teams from the Delta Amacuro department of the Ministry of Health and Social Welfare, and vaccines are provided free of charge.

### Population

In May 2012, PCV13 was first introduced in nine communities in the Orinoco Delta by means of an efficacy study [12]. During this study, PCV13 acceptance or refusal was recorded. The target group for this qualitative study consisted of primary caregivers of children aged 6 weeks– 6 months residing in one of nine communities. These mothers were approached six months later, in November 2012. By then, three consecutive doses of PCV13 had been offered to their children as a primary series, following Centers for Disease Control and Prevention (CDC) guidelines [13].

### Data collection

The research consisted of interviews carried out in caregivers’ homes and lasting approximately 30 minutes. Interviews were held in Spanish or with a local translator in the Warao language. An interview guide was used for each interview. To compose the interview guide, we performed an electronic search using the TRIP database, Cochrane Library and Pubmed with the key words ‘vaccination’, ‘immunization’, ‘acceptance’, ‘refusal’, ‘coverage’, ‘decisions’, ‘attitudes’, ‘perceptions’ and ‘knowledge’, ‘indigenous’, in various combinations. Additional studies were identified by searching the reference lists from existing articles. We also based the interview guide on our own observations and experiences. The interview guide contained questions about general knowledge and risk perception of diseases in general and diseases related to *Streptococcus pneumoniae* specifically as well as motivations for accepting or declining vaccines in general and PCV13 specifically. All questions were open-ended. The interviews were recorded and each interview was typed out ad verbatim.

### Data analysis

We followed the six phases of thematic analysis as described by Braun and Clarke [14]. Two of the authors transcribed and familiarized themselves with the data. Initial codes were then assigned to segments of the raw data using the qualitative data analysis software MAXQDA (VERBI Software, Berlin, Germany). The two researchers then read coded segments together in order to reach a consensus about the applied codes. The different codes were sorted into potential overarching themes by both researchers separately. Following this phase, discussions about the fit and validity of candidate themes and sub-themes were held. Finally, the authors agreed on final key themes. These themes were repeatedly checked against the transcripts in order to identify patterns. Interpretation of themes was informed by the literature, objectives of the study, and additional discussions with two other authors with ample working experience in the Orinoco Delta.

### Ethical considerations

The natures and objectives of the study were explained in Spanish and/or in their native language and primary caregivers provided written informed consent. The study was approved by the ethical committee of the Instituto de Biomedicina and by community leaders (consejos comunales).

## Results

We performed a qualitative survey exploring parental decision-making about childhood vaccines. In total, 67 caregivers residing in the nine study communities (Fig 2) were eligible. Of these, 42 (63%) had accepted vaccination with PCV13 while 25 (37%) had declined one, several or all vaccines. Approximately half of the caregivers in each group were interviewed, resulting in 20 and 11 interviews respectively. Of the 11 vaccine-declining mothers, 9 had declined all vaccines and 2 had accepted the first dose but refused follow-up doses. Of the total of 31 included caregivers, 30 were the mother of the child and one primary caregiver was the child’s grandmother.

### Characteristics of the study population and vaccine decision-making processes

The majority (55%) of the respondents were housewife. Others produced handicrafts or worked in the paddy fields for taro (tuber) cultivation. Almost one-third of the mothers did not have any education and roughly half only attended elementary school. Most had large families; 13 caregivers (42%) had 3 to 5 children and 9 (29%) had more than 5 children. All caregivers attended Western health care facilities (community posts or hospitals) in case of illnesses and over two thirds (68%) also visited traditional healers. Characteristics of the study population are summarized in Table 1. The 20 vaccine-accepting respondents all said they accepted vaccination because vaccines either prevent diseases (n = 12) or prevent diseases from deteriorating (n = 7) or cure diseases (n = 1). In addition, some (n = 3) said that discussing the matter with family or community members helped them to make the decision to vaccinate their children. However, overall, fathers or other family members only seemed to play a minor role in decisions regarding vaccine acceptance. Of the total number of 31 vaccine acceptors and refusers, 28 mothers (90%) did not consult others in order to make a decision. Traditional healers or religion did not seem to play a role in the decision regarding vaccination or non-vaccination of children. Only two mothers had asked a traditional healer for his opinion concerning childhood vaccination and none of the caregivers mentioned her religious background in relation to vaccine practices.

**Table 1:** Characteristics of study population of Warao Amerindian caregivers in Venezuela.

Characteristic	N (%)
Age, median (IQR)	28 (26–34)
Occupation, n (%)	
Housewife	17 (55)
Ocumo Farmer	4 (13)
Handcrafts	5 (16)
School Worker	2 (7)
Teacher	1 (3)
Government Worker	1 (3)
Home worker	1 (3)
Education, n (%)	
None	10 (32)
Attended Primary School	14 (45)
Attended High School	7 (23)
Marital status, n (%)	
Married	16 (52)
Unmarried	7 (23)
Widowed	1 (3)
Cohabited	7 (22)
Number of children per mother, n (%)	
1 or 2	9 (29)
3 to 5	13 (42)
5 or more	9 (29)
Sex of child aged 6 weeks—6 months, n (%)	
Male	14 (45)
Female	17 (55)
Religion, n (%)	
None	9 (29)
Christian	22 (71)
Health facilities visited in case of illness	
Western medicine	31 (100%)
Traditional healer	21 (68%)
Literate, n (%)	
Yes	17 (55)
No	14 (45)
Vaccine acceptance, n (%)	
Accepted all vaccines	20 (65)
Refused 1 or more vaccines	11 (35)
Community, n (%)	
Araguabisi	5 (16)
Araguaimujo	7 (23)
Arature	3 (10)
Bonoina	3 (10)
Guayaboroina	2 (6)
Ibaruma	2 (6)
Jobure de Curiapo	5 (16)
Merejina	1 (3)
Winikina	3 (10)

Interestingly, all 11 vaccine-declining mothers also believed that vaccines helped to prevent (deterioration of) diseases. Despite this general recognition of vaccine health benefits, none of the 31 participants was aware of the immunological mechanism of vaccines, i.e. to trigger the immune system to develop a response against a killed or inactivated pathogen. The most widely recognized vaccines were measles (mentioned by 52%), varicella (26%), pertussis (16%), tetanus (13%) and yellow fever (13%). Only two mothers mentioned a pneumonia vaccine, while PCV13 had been offered to all mothers only six months before. Over one third of the respondents (35%) mentioned that vaccines were also administered against a variety of non-specific conditions, including vomiting, fever, diarrhea, headache and arthritis. Caregivers generally accepted ‘all’ or ‘no’ vaccines and none of the respondents reported refusal of specific vaccines.

Reasons to refuse vaccination were discussed with vaccine-declining mothers. Also, vaccine-accepting mothers were asked about situations in which they would not vaccinate their children. Table 2 sums up the motives for (potential) vaccine refusal in the study population. Three main themes became evident: 1) vaccine refusal due to fear of side effects, 2) vaccine refusal due to perceived limited vaccine tolerance of young and sick children and 3) the empirical concept that side effects of vaccines are diseases.

**Table 2:** (Potential) vaccine refusal reasons mentioned by all and vaccine-declining Warao Amerindian caregivers in Venezuela.

**All caregivers (n = 31)**	**Number (% of total interviews)**
If an illness is present at the moment of vaccination	17 (55)
Fever	15 (48)
Diarrhea	7 (23)
Runny nose	4 (13)
Vomiting	2 (6)
Appearance of teeth	1 (3)
Headache	1 (3)
Abdominal pain	1 (3)
Undefined	4 (13)
Concerns about side effects	28 (90)
**Vaccine-declining caregivers (n = 11)**	**Number (% of 11 interviews)**
Side effects do not outweigh benefits	11 (100)
Child is too young to vaccinate	4 (36)
Same vaccine should not be given twice	1 (9)

### Fear of adverse events leading to refusal of vaccines

All 11 vaccine-declining mothers mentioned concerns about unwanted effects as the main reason for refusing vaccination. In addition, 17 of the vaccine-accepting caregivers also mentioned a wide variety of symptoms and diseases caused by vaccination, but they stated that the beneficial effects of vaccination outweighed these side effects. The potential unwanted effects and the frequency with which they were reported are listed in Table 3. Notably, not only known side effects such as fever were mentioned. Diarrhea was also a side effect many (n = 7) mothers worried about. Four mothers even said that children could die because of vaccination.

**Table 3:** Effects of vaccination as mentioned by 28/31 Warao Amerindian caregivers in Venezuela.

Effect	Number of caregivers mentioning effect (% of total interviews)
Fever	26 (84)
Swollen skin	8 (26)
Diarrhea	7 (23)
Illness	6 (19)
Death	4 (13)
Vomiting	4 (13)
Dermatitis	4 (13)
Pain	2 (6)
Impaired walking ability	2 (6)
Harm in general	1 (3)
Abscess	1 (3)
Tumor	1 (3)

*Vaccination teams have come here on several occasions but I never choose to vaccinate my children. We fear vaccines, children get fevers, diarrhea and they can die too due to vaccination. And now all my children are healthy! […] They (the vaccination teams) say that the fever goes away, but it doesn’t always go away and if fevers get really high some children die. My children are growing up being well, we also grew up without vaccinations*.

(Mother of 7 children, 38 years old)

Side effects were commonly ascribed to all vaccines, although one mother mentioned specifically that the pentavalent vaccine causes fever. Four vaccine-declining mothers said they would consider having their children vaccinated if antipyretics would be offered upon vaccine administration. Their fear that vaccine side effects would develop in the absence of access to medical care made them refuse vaccination altogether. Warao families generally live far from hospital facilities and boat trips of up to seven hours have to be made to reach the nearest medical post. In addition, these medical posts are often under-equipped and short of medicine.

*If children are vaccinated, they immediately get a fever, so they have to give us medication for these diseases. Because of the fever, the child gets hot from the inside, in its belly, and therefore also starts to vomit and gets diarrhea […] The people that vaccinate children do not provide us with medicine for the diseases caused by these vaccines and if I go to a medical post they can’t help me either, that’s when I get mad*.

(Mother of 10 children, 43 years old)

Three of the vaccine-declining mothers said they would consider vaccinating their children if given a more thorough explanation about the vaccine at the moment of administration. These mothers felt that vaccines were often given without a conversation with the primary caregiver which made them unwilling to participate.

### Perceived limited vaccine tolerance of young and sick children

More than half of the mothers (55%) said they would not vaccinate their children when they are ill at the moment the vaccine is offered. The definition of ‘illness’ that would lead to refusal of vaccines included fever, diarrhea, flu, vomiting, the appearance of teeth, headache and abdominal pain (Table 2). The general opinion was that the child should be strong, and thus not suffer from any of the symptoms mentioned, in order to be able to handle a vaccination.

In addition, a common perception (mentioned by 19% and 36% of all and vaccine-declining respondents respectively) was that young children were too vulnerable or weak to be vaccinated. Older children were regarded to be stronger and less prone to side effects. Two mothers explicitly mentioned that they did tolerate oral vaccines for young children.

*To my youngest child, they only gave him the drops in his mouth, since he is too young, he cannot be injected. Because if it hurts him and he gets sick, we cannot get him to a hospital. We do not have a motorboat to get him to a hospital*.

*(Mother of 5 children, 31 years old)*.

In Warao communities, the age of children is often unknown and birthdays are not celebrated. Caregivers that refused vaccines for young children could not indicate at what age they would have their children vaccinated, some said the child would have to grow ‘a bit bigger’, some said that it would take ‘several months’ and some pointed to an older sibling as having the appropriate age to be vaccinated. In addition to the perception that young children were too vulnerable to be vaccinated, notably, 9 mothers (29%) mentioned that young children are the ones that are most susceptible to (severe) diseases.

### Symptom-based disease interpretation

The distinction between ‘symptoms’ and ‘diseases’ was not made by any of the primary caregivers. When parents were asked about diseases in general, the ‘diseases’ that were mentioned first in more than half of the mothers (n = 18, 58%) were either diarrhea (39%) or fever (19%). Some mothers described clusters of symptoms that could occur together (e.g. diarrhea, vomiting and fever), but these were generally regarded to be three separate diseases that could either attack in varying combinations or individually. Fever and diarrhea were also among the most commonly described effects of vaccination (mentioned by respectively 84% and 23% of the 31 caregivers, Table 3) and also then described as ‘diseases caused by vaccination’. The term ‘(side) effects’ of vaccination was not used by any of the Warao mothers and all vaccine-declining mothers regarded the fever caused by vaccination to be a disease in itself warranting adequate treatment, but even then could harm their children.

*When they vaccinate our children, they develop a fever, that’s why we do not want to vaccinate them. After the vaccination teams leave, the vast majority of our children is ill, so we prefer not vaccinating them. We really fear the vaccines because of the fever […] I have not been vaccinated and I live in peace: working, eating, ….I do not suffer from diarrhea or fevers*.

(Mother of 4 children, 27 years old)

## Discussion

This study demonstrates which ideas about vaccination and diseases influence the acceptance or refusal of vaccines in Amerindian mothers of young children in the Orinoco Delta in Venezuela. All participants, both vaccine-accepting and vaccine-declining, claimed to see the benefits of vaccination on the grounds that it “prevents diseases” or “prevents the deterioration of diseases”. However, three main themes were identified that negatively influenced vaccine acceptance. Our findings provide starting points for the improvement of vaccine education strategies.

Concerns about adverse events are also among the main barriers to vaccination in other South American countries [10] as well as in low- and middle-income countries in other parts of the world [7]. In fact, even in Western populations concerns about vaccine safety and serious side effects are among the main reasons for delay or refusal of childhood vaccines [15]. The perception that unwanted symptoms due to vaccination are diseases warranting adequate treatment has, to our knowledge, not been previously described in indigenous South American populations. Dugas et al. also described a major role of the empirical concept of childhood disease in low vaccination coverage rates among ethnic groups in Burkina Faso, Africa [16]. In concordance with their study, symptoms/diseases that were most often mentioned by Warao mothers resembled the frequently named side effects of vaccination, with most importance assigned to fever and diarrhea. The similarity between these symptoms/diseases and vaccine adverse effects is their high prevalence of occurrence in these rural villages and the ease with which they can be recognized. In the absence of biomedical screening tests, it is not surprising that it is understood by the Warao people that the fever or diarrhea *are* the diseases, rather than symptoms of an underlying disease. In addition, the finding that one third of the respondents mentioned that vaccines were given in order to prevent non-specific symptoms, including fever and diarrhea, may further limit vaccine acceptance. Since most vaccines target a single disease, their perceived effectiveness may be disappointing when a fully vaccinated child develops diarrhea or fever, especially if this is the result of the side effects of vaccination.

The unclear hierarchical distinction between disease symptoms and processes (e.g., diarrhea versus parasitic disease) within the biomedical system has also been described in other South American indigenous groups [17, 18]. Also, the concept of ‘symptom-oriented’ diseases in Warao people has been identified in anthropological studies [19, 20]. The basis for this perception probably lies in the idea that symptoms are caused by weakness of one or more of the four souls the Warao people believe are present in each human being. Specific combinations of ‘soul weaknesses’ are thought to cause specific symptoms with a maximum of three symptoms occurring together [20]. The finding that this symptom-based disease interpretation among Warao people affects concerns about side effects and vaccine acceptance is new and warrants further exploration. It may be necessary to specifically address the topic of weaknesses related to vaccine side effects through vaccine education programs.

Thoughts around weakness and vulnerability to vaccine side effects and disease may also underlie the common idea that young and sick children are not strong enough to handle vaccination. In rural Bangladesh, mother’s perception of small infant size at birth was negatively associated with timely vaccination [21]. Other studies also report lower vaccine coverage in young children in relation to parental beliefs including young children being too little, immature and fragile to handle immunizations [22, 23]. This phenomenon could explain why only 63% of the caregivers of young children ≤6 months had accepted vaccination while PCV13 in this context was overall accepted by 84% of Warao mothers of children <5 years in the study area [24]. The fear that young children would suffer too much from vaccination was accompanied by the perceived vulnerability of these children to (severe) diseases. Mortality rates in Warao children are indeed extremely high in the first year of live, when 54% of childhood deaths occur [11]. The observation that young children are most likely to die when they get ill seems to fuel the concern that vaccine adverse events may more severely harm these vulnerable children. This fear is related to thoughts around the origin of diseases but also to a lack of information concerning the actual effect of vaccines on the body or the potential side effects. The conflict between biomedicine and local understandings has been identified as a barrier to vaccination in ethnic people in Africa as well [25].

Half of the vaccine-declining mothers would be willing to have their children vaccinated if more information and antipyretics would be offered. Whereas in other societies parents often have access to a bulk of available information, e.g. through the Internet [26], Warao generally lack Internet, television, radio or newspapers. For information regarding vaccination, they rely on community members and the vaccination teams themselves. Vaccination teams are not community members; they enter the Orinoco Delta by boat and generally stay for only a couple of days. Cold boxes filled with big ice blocks are used to keep vaccines cold during transportation. The outside temperature of >25°C limits the lifetime of ice blocks. Boat trips of around 6–10 hours (depending on the material of which the boat is made, the horsepower of the engines, and on how heavily the boat is loaded) are made to reach the villages in Antonio Diaz from the mainland. In these boats, 200-liter barrels of gasoline are shipped from the mainland to the River Delta in order to reach as many communities as possible during one trip. There is thus limited space onboard for cold boxes carrying extra ice. These logistical challenges lead to a strategy aimed at reaching as many communities as possible in as little time as possible. It is, however, unsatisfactory that one third of Warao mothers of young children refuses vaccination when communities are reached despite these logistical problems. We recommend that vaccine education programs are separated from vaccination campaigns so that time and effort can be put into addressing the concerns of caregivers. In particular, vaccine education programs that precede an upcoming vaccination campaign may be effective. A recently published systematic review shows that multicomponent and dialogue-based interventions to improve vaccine uptake perform best [27]. There is, however, limited evidence for the effectiveness of interventions focused on raising knowledge and awareness. It thus seems advisable to discuss proposed intervention strategies with community members before implementation. In addition, quantification of the impact of educational interventions is necessary to determine their effectiveness. In addition to educational interventions, the standard distribution of antipyretics together with vaccine administration and explanation about its use may increase vaccine compliance.

Other studies, including from South American indigenous populations, have documented that practice of traditional medicine, religious beliefs or a lack of faith in Western medicine may negatively affect decisions around health care and vaccination acceptance [16, 28–31]. Although the majority of Warao caregivers visited traditional healers (68%) and were Christians (71%), a major role of these factors in vaccine decision-making was not identified. Over the past 20 years, acculturation has led to increased acceptance of Western medicine, as can be seen from the finding that all mothers claimed to visit Western health care facilities. Educational programs can use this acknowledgment of the value of Western medicine in general to reassure Warao people that vaccines have proven both successful and safe.

A strength of our study is the prospective recording of vaccine acceptance or refusal. Self-reported vaccine uptake limits the reliability of many studies addressing factors underlying parental vaccination decisions [32]. A limitation of this method was that we had to re-locate specific caregivers with recorded information on vaccine refusal or acceptance rather than interview those women present when we performed the current study. This, together with logistical constraints, was the main reason that only half of the eligible caregivers were interviewed. Warao people are semi-nomadic and often migrate temporarily from their communities for agricultural purposes. In addition, because only two women were included that refused several but not all vaccine doses, we were not able to distinguish possible differences between reasons for total or partial / temporal refusal.

We did not interview fathers or other family members and can thus not reflect on fathers’ attitudes and beliefs concerning vaccination. However, primary caregivers in Warao families are usually mothers, as illustrated by the observation that 28/31 mothers stated they made decisions regarding vaccination themselves, regardless of the input of family members.

## Conclusion and Recommendations

This study provides a perspective into the low rate of vaccine acceptance among Warao Amerindian caregivers of young children in Venezuela. Three important findings emerged. First, fear of side effects was the most important immunization barrier. Second, these side effects were regarded as diseases for which they felt treatment should be offered. Finally, according to caregivers, children needed a certain level of strength to be able to handle vaccination leading to limited vaccine acceptance in young children.

Since all mothers did believe vaccines could reduce disease incidence, it is important to set up vaccine education programs focused on the explanation of the origin of side effects, the vulnerability of children to these effects in relation to age and their self-limiting nature.

## Supporting Information

S1 File Topic list used by the interviewers (original in Spanish). (DOCX)

S2 File Topic list used by the interviewers (translation in English). (DOCX)
