# Supplementary material for: Childhood Vaccine Acceptance and Refusal among Warao Amerindian Caregivers in Venezuela; A Qualitative Approach
Source: PLoS One. 2017 Jan 20;12(1):e0170227. doi: 10.1371/journal.pone.0170227 (PMC5249092; doi:10.1371/journal.pone.0170227)
Supplement: S1 File — (DOCX) [file pone.0170227.s001.docx]

**Childhood (pneumococcal) vaccine acceptance and refusal among Warao Amerindian caregivers in Venezuela**

| Fecha del cuestionario: | Educación de la madre: |
| --- | --- |
| Nombre de la madre: | ¿Sabe leer y escribir?: |
| Fecha de nacimiento de la madre: | ¿Estás casada?: |
| Comunidad: | ¿Qué trabajo haces?: |
| Número de hijos: | Trabajo del marido: |
| Sexo del niño (6s - 6m): | Educación del marido: |
| Fecha de nacimiento del niño: | Religión: |

**Conocimiento general:**

- ¿Cuáles son las causas de enfermedades? ¿Cómo se puede enfermar un niño o un adulto?
- ¿Hay cosas que uno puede hacer para no enfermarse o para que no se enfermen las personas?
- ¿Cuál es el objetivo de las vacunas? ¿Para qué se vacunan las personas?
- ¿Cuáles son las ventajas de las vacunas? ¿Cuáles son las desventajas o efectos desagradables?
- ¿Tiene sentido colocar la misma vacuna varias veces (refuerzos)?
- ¿Cuándo no se puede vacunar a un niño? ¿Cuáles son las razones para que la mamá decida que no quiere vacunar a un niño en el momento que hay un equipo de vacunación?
- ¿Usted ha escuchado algo sobre la vacuna contra la neumonía?
- ¿Cuáles vacunas conoce?

**Determinación de riesgos:**

- ¿El riesgo o chance de que se enferme su hijo es grande o pequeño? ¿Es muy probable que se enferme su hijo o no se enferma fácilmente?
- ¿Conoce la neumonía/la enfermedad que da el pecho trancado? ¿Cuáles son los síntomas?
- ¿Conoce la otitis/la enfermedad de los oidos? ¿Cuáles son los síntomas?
- ¿Conoce la meningitis/la enfermedad que da mucha debilidad con fiebre alta? ¿Cuáles son los síntomas?
- ¿Quiénes se pueden enfermar de neumonía/otitis/meningitis? ¿Esas son enfermedades graves?
- ¿Alguno de sus hijos en algún momento ha sufrido de neumonía/otitis/meningitis?
- ¿Usted en algún momento ha sufrido de neumonía/otitis/meningitis? ¿Y otro adulto?
- ¿Cómo se trata un niño con neumonía/otitis/meningitis?
- ¿Con cuáles enfermedades va al hospital/brujo?

**Razones para aceptar o declinar vacunas:**

- ¿Usted por lo general vacuna a su hijo/hija cuando vienen los equipos de vacunación?
- ¿Por qué normalmente acepta o no acepta una vacuna?
- ¿Cuál es el beneficio de colocar vacunas/la vacuna contra la neumonía?
- Vacunando a todos los niños de una comunidad contra una enfermedad, ¿La enfermedad le puede dar después a quienes no estén vacunados? ¿Usted cree que es verdad que se puede proteger a toda la comunidad contra enfermedades cuando se vacuna solamente a los niños?

**Influencias sociales:**

- ¿Hay alguien más que decide con usted si acepta o no una vacuna para sus hijos? O que le da consejos sobre eso.
- ¿Cómo supo sobre las vacunas? ¿Quiénes les han informado sobre las vacunas?
