# Supplementary material for: Childhood Vaccine Acceptance and Refusal among Warao Amerindian Caregivers in Venezuela; A Qualitative Approach
Source: PLoS One. 2017 Jan 20;12(1):e0170227. doi: 10.1371/journal.pone.0170227 (PMC5249092; doi:10.1371/journal.pone.0170227)
Supplement: S2 File — (DOCX) [file pone.0170227.s002.docx]

**Childhood (pneumococcal) vaccine acceptance and refusal among Warao Amerindian caregivers in Venezuela among the Warao Amerindians in Venezuela**

| Interview date: | Education mother: |
| --- | --- |
| Name mother: | Do you know how to read and write: |
| Birth date mother: | Are you married: |
| Community: | What kind of work do you do?: |
| How many kids do you have: | Work husband: |
| Gender child (6w - 6m): | Education husband: |
| Birth date child: | Religion: |

**General knowledge:**

- What causes diseases? How does a child or adult get sick?
- What can you do to prevent people from getting sick?
- What is the objective of vaccination. Why do people vaccinate?
- What are benefits of vaccines? What are disadvantages of vaccines?
- How do you think about application of the same vaccine multiple times (boosters)?
- When is it not allowed to vaccinate children? What are reasons to refuse a vaccination when a vaccination team arrives?
- Have you ever heard about a vaccine against pneumonia?
- Can you name the vaccines you know?

**Risk determination:**

- Is the risk of your child getting sick a great or small risk? Is it likely that your child will become ill or does he/she not get sick easily?
- Are you familiar with pneumonia/the illness that affects your lungs? Symptoms?
- Are you familiar with otitis/the illness that affects your ears? Symptoms?
- Are you familiar with meningitis/the illness that results in weakness and high fever? Symptoms?
- Who are susceptible for pneumonia/otitis/meningitis? Are these serious diseases?
- Has one of your children at any time ever suffered from pneumonia/otitis/meningitis?
- Have you yourself at any time ever suffered from pneumonia/otitis/meningitis? Other adults you know?
- How do you treat a child that suffers from pneumonia/otitis/meningitis?
- With what diseases do you go to the hospital or witch doctor?

**Reasons for accepting or refusing vaccines:**

- Do you generally accept vaccines for your son or daughter when vaccination teams arrive?
- Why would you accept or refuse a vaccine?
- What is the benefit of vaccination/applying the pneumococcal vaccine?
- If all children in a community are vaccinated against a disease, can other people who are not vaccinated still contract the disease afterwards? Do you think that vaccination of some may be helpful in the protection of others or the whole community against diseases?

**Social influences:**

- Is there anyone else who decides whether you will or will not vaccinate, or someone you ask for advice on this matter?
- How do you know about vaccines? Who has informed you about them?
